# *MOS* is a novel genetic marker for human early embryonic arrest and fragmentation

**DOI:** 10.15252/emmm.202115323

**Published:** 2021-11-22

**Authors:** Lei Wang, Qing Sang

**Affiliations:** ^1^ Institute of Pediatrics Children’s Hospital of Fudan University the Institutes of Biomedical Sciences, and the State Key Laboratory of Genetic Engineering Fudan University Shanghai China

**Keywords:** Genetics, Gene Therapy & Genetic Disease, Neuroscience, Organelles

## Abstract

Early embryonic arrest and fragmentation (EEAF) is a common phenotype observed in *in vitro* fertilization (IVF) or intracytoplasmic sperm injection (ICSI) cycles. The phenotype causes female infertility and recurrent failed IVF/ICSI attempts. However, the molecular mechanisms behind EEAF remain largely unknown. In this issue of *EMBO Molecular Medicine*, Zhang *et al* (2021) present the novel causative gene *MOS* in patients with the EEAF phenotype. The relationship between *MOS* variants and human EEAF is comprehensively established through a series of *in vitro* and *in vivo* experiments, thus clarifying the role of MOS during human oocyte maturation and early embryo development. These findings suggest that *MOS* is a new diagnostic marker of EEAF and is a potential therapeutic target for treatment of EEAF patients.

## Graphical abstract

L. Wang and Q. Sang discuss the study by Zhang *et al* (in this issue of *EMBO Mol Med*) that reports Moloney sarcoma oncogene (MOS) as a novel genetic marker for early embryonic arrest and fragmentation.

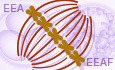

Infertility affects millions of people worldwide and can be caused by a number of different factors such as environmental, endocrine and genetic factors, and aging (Bala *et al*, 2021). Assisted reproductive technology has helped many couples to have their own children, and it is estimated that more than 8 million babies have been born using the technology (Fauser, 2019). In the initial step, oocytes undergo the process of maturation to become metaphase II (MII) oocytes. Only oocytes at this stage can be fertilized and start the first cleavages. The resulting embryos are then cultivated *in vitro* to develop into eight‐cell embryos or blastocysts, at which point they can be transferred into the uterus to establish pregnancy. Abnormalities in any step of this procedure will result in failure of *in vitro* fertilization (IVF) or intracytoplasmic sperm injection (ICSI) attempt. In the clinic, many patients undergo several rounds of unsuccessful IVF/ICSI cycles due to arrest or abnormalities of oocytes or early embryos without knowing the exact molecular reasons for such failure. This leads to great economic and psychological burdens in these patients, and thus understanding the molecular basis of infertility is at the essence of precision medicine and future potential treatments in reproductive medicine.

In 2016, the first pathogenic gene *TUBB8* was identified to be responsible for oocyte metaphase I (MI) arrest, which suggested that single mutant genes could play important roles in abnormalities of human oocyte maturation (Feng *et al*, 2016). Several other mutant genes have since been shown to cause abnormalities in the process of oocyte maturation, fertilization, and early embryonic development (Sang *et al*, 2018, 2019, 2021). These mutant genes cause a variety of phenotypes, including oocyte germinal vesicle (GV) arrest, MI arrest, fertilization failure, oocyte death, zygotic cleavage failure, and early embryonic arrest. These findings suggest that a few Mendelian phenotypes are hidden within the process of oocyte maturation and early embryonic development.

In the clinic, embryonic fragmentation is a common phenomenon that results in early embryo arrest or low embryo quality. Previous reports mainly focused on sub‐cortical maternal complex‐related genes as well as some *TUBB8* variants that cause embryonic arrest without showing fragmentation (Fig 1). In contrast, the molecular mechanisms behind embryonic fragmentation are largely unknown.

**Figure 1:**
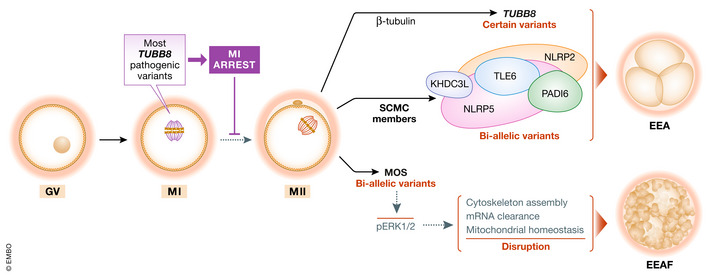
A schematic showing the reported genetic determinants of human EEA and EEAF TUBB8 is a β‐tubulin isotype and plays an important role in human oocyte spindle assembly. Most *TUBB8* pathogenic variants cause the phenotype of oocyte MI arrest. Certain heterozygous or bi‐allelic variants in *TUBB8* have been shown to result in early embryonic arrest (EEA). Previous articles have reported that bi‐allelic variants in genes encoding proteins of the subcortical maternal complex (SCMC), including *NLRP2*, *NLRP5*, *TLE6*, *PADI6*, and *KHDC3L*, are responsible for EEA. MOS activates the ERK signaling cascade to maintain oocyte MII arrest. Bi‐allelic pathogenic variants in *MOS* inactivate the MOS‐ERK pathway and therefore cause disruption of cytoskeleton assembly, mRNA clearance, and mitochondrial homeostasis, which accounts for the phenotype of human EEAF.

To explore the pathogenesis of human early embryonic arrest (EEA), especially in cases with the combined phenotype of early embryonic arrest and fragmentation (EEAF), Zhang *et al* (2021) performed whole‐exome sequencing in a cohort of EEAF patients and identified the novel mutant gene *MOS* in three independent families following a recessive inheritance pattern. All patients carrying bi‐allelic pathogenic variants of *MOS* exhibited the same EEAF phenotype.

MOS is a serine/threonine protein kinase that activates the ERK pathway, and it is highly and specifically expressed in vertebrate oocytes and functions as a cytostatic factor to maintain oocyte MII arrest. Although the function of MOS has been clarified in the oocytes of several vertebrate species, the exact role of MOS in human oocytes is unknown. The identification of MOS variants by Zhang *et al* (2021) in human EEAF patients is thus a breakthrough in our understanding of the physiological function of MOS in human oocytes and early embryos.

The authors first present the protein dynamics of the MOS‐ERK pathway under physiological conditions in human oocytes, zygotes, and early embryos, suggesting the critical maternal effect role of MOS in human MII oocytes. The authors then present the pathogenicity of the identified variants through a series of *in vitro* experiments, and they show that MOS variants lead to ERK inactivation both in HEK293 cells and mouse oocytes and that such variants cannot reverse the pERK1/2 level upon MOS insufficiency in mouse oocytes, thus proving the functional impairment of patient‐derived protein variants. As an explanation for the phenotype of fragmentation, the authors suggest that interfering with the MOS‐ERK pathway significantly weakens F‐actin intensity and causes α‐tubulin instability in oocytes, which at least partially accounts for the generation of severe embryo fragmentation (Fig 1).

It has been previously demonstrated by the authors that ERK1/2 regulates maternal mRNA decay in mouse oocytes (Sha *et al*, 2017). Thus, the authors wanted to know whether MOS, an upstream molecule of ERK1/2, participates in the maternal mRNA clearance in human oocytes. By comparing the expression of gene transcripts in MII oocytes between healthy controls and patients with the Asn95Lys MOS variant, the authors demonstrated that inactivation of the MOS‐ERK pathway affects maternal mRNA clearance during human oocyte maturation. Because blocking of mRNA clearance results in early embryonic arrest (Zhao *et al*, 2017), the disruption of mRNA clearance in oocytes with MOS variants might therefore be an explanation for the phenotype (Fig 1).

Finally, GO analysis revealed that genes associated with the biological processes of mitochondrial function were severely dysregulated. The authors then confirmed the mitochondrial dysfunction by measuring mitochondrial distribution, membrane potential, and ATP production. Mitochondrial activity is an important regulator of oocyte quality and is associated with embryo integrity (Harvey, 2019); thus, mitochondrial dysfunction may be another contributor to embryo fragmentation in patients with *MOS* mutations (Fig 1).

In this study, the authors have established the direct relationship between *MOS* mutations and human EEAF. *In vivo* functional studies using control and patient oocytes strengthen the causality of the identified variants and uncover the physiological function of MOS in human oocytes. This finding provides a diagnostic marker for EEAF and indicates that screening for pathogenic mutations in genes of the MOS‐ERK pathway may provide further understanding of the pathogenesis of human EEAF.

To better understand the role of MOS in human fertility, additional studies are needed in the future. For example, apart from EEAF, an important question is whether MOS insufficiency will lead to other phenotypes, including oocyte maturation arrest, fertilization failure, and recurrent miscarriage. The relationship between disrupted mRNA clearance and cytoskeleton assembly defects also deserves further investigation. In addition, it will be valuable to explore therapeutic strategies by using transgenic mice in which wild‐type *Mos* is replaced with mutant human *MOS*. These studies will help us to better understand the pathogenic mechanisms of abnormalities in human early embryonic development and will provide potential therapeutic treatments for these patients in the future.
